# Genome sequence of the pathogenic *Verticillium longisporum* A1D3 strain PD614 (IPP1195) isolated from oilseed rape in Germany

**DOI:** 10.1128/mra.00171-26

**Published:** 2026-07-10

**Authors:** Annette Pfordt, Dominic Simm, Birger Koopmann, Andreas von Tiedemann, Martin Kollmar

**Affiliations:** 1 Plant Pathology and Crop Protection Division, Department of Crop Sciences, Georg-August-University Göttingen, Göttingen, Germany; 2 GOENOMICS GmbH, Göttingen, Germany; University of Maryland School of Medicine, Baltimore, Maryland, USA

**Keywords:** genome sequence, *Verticillium longisporum*

## Abstract

We present the genome assembly and annotation of the pathogenic *Verticillium longisporum* strain IPP1195 (PD614), a member of the A1D3 lineage isolated from oilseed rape in Germany. This genome provides a reference for pathogenic A1D3 isolates and supports comparative studies of virulence and hybrid genome evolution.

## ANNOUNCEMENT

Species of the genus *Verticillium* are soil-dwelling ascomycete fungi that infect plants through the roots and spread systemically via the xylem. Among them, *Verticillium longisporum* is a hybrid species with a predominantly Brassicaceae-associated lifestyle and is a major causal agent of *Verticillium* wilt in oilseed rape (*Brassica napus*). Comparative genomic and phylogenetic studies have demonstrated that *V. longisporum* consists of three principal hybrid lineages—A1D1, A1D2, and A1D3—which differ in genomic composition, host interaction, and pathogenic potential (1, 2). In contrast to many A1D1 isolates that show variable aggressiveness, strains assigned to the A1D3 lineage are consistently associated with pathogenicity on oilseed rape and related hosts (2, 3).

Here, we report the genome assembly and annotation of *V. longisporum* strain IPP1195, also referred to as MD73 or PD614. The isolate was obtained from oilseed rape in Germany in 2011 and was received in 2015 from Patrick Inderbitzin, as mentioned in reference 1. It has been stored as a lyophilizate at 8°C in a cooling chamber.

DNA extraction and sequencing were performed by BMKGene (Münster, Germany). HMW genomic DNA was extracted from 0.5 g of mycelium using a CTAB-based extraction method. In this protocol, sodium acetate was used instead of sodium chloride; it was omitted from the extraction buffer and applied during the precipitation step. For library preparation, 0.75–1.5 μg of genomic DNA was mechanically sheared using g-TUBE devices and subsequently purified with AMPure PB beads according to the manufacturer’s protocol. No size selection was applied. Sequencing libraries were prepared using the SMRTbell Prep Kit 3.0 and sequenced on the PacBio Revio, generating long-read data corresponding to an estimated genome coverage of approximately 46.6× (479,626 reads, 3,688,910,268 sequenced bases, and a read N50 of 9,999).

*De novo* genome assembly was performed using Hifiasm v0.25.0-r726 (4), with parameters --primary -l0 (default parameters were used except where otherwise noted) producing a chromosome-scale draft assembly. The NCBI Foreign Contamination Screen v0.5.5 (5) was used to detect contaminations. A representative mitochondrial contig was generated with MitoHiFi v3.2.1 (6). The assembly comprised a total length of 72.77 Mb and a GC content of 53.42% and consisted of 63 contigs, with 16 contigs above 1 Mb and an N50 value of 5.09 Mb.

Genome completeness was evaluated using BUSCO v5 (7) with the glomerellales_odb12, yielding a completeness score of 99.18% when considering complete single-copy and duplicated orthologs, indicating comprehensive recovery of conserved fungal genes. Structural annotation was conducted using Mendle Analytics, integrating protein homology mapping, transcript-based evidence where available, and *ab initio* gene prediction models trained on *Verticillium* genomes. This annotation strategy resulted in 19,445 predicted protein-coding genes, accompanied by a set of noncoding RNA loci.

The genome sequence of strain IPP1195 provides a valuable reference for the pathogenic A1D3 lineage of *V. longisporum*. This resource enables comparative analyses between pathogenic and less aggressive lineages and supports future studies on virulence determinants, hybrid genome evolution, and host adaptation in oilseed rape-infecting *Verticillium* populations.

## Data Availability

The Whole Genome Shotgun projects have been deposited at DDBJ/ENA/GenBank under accession number JBUBAF000000000. The version described in this paper is version JBUBAF010000000. The BioProject is available under accession number PRJNA1375490, and the SRA is available under accession number SRR36998063. Annotation is available via Figshare (10.6084/m9.figshare.31655614).
